# Long-time irradiation effect on corrosion behavior of aluminum alloy in pool water of low-power research reactor

**DOI:** 10.1038/s41598-023-44287-0

**Published:** 2023-10-09

**Authors:** Mojtaba Bagherzadeh, Meysam Karimi, Mohammad Hosein Choopan Dastjerdi, Mohsen Asadi Asadabad, Javad Mokhtari, Afshin Babanejhad

**Affiliations:** Reactor and Nuclear Safety Research School, Nuclear Science and Technology Research Institute, Tehran, Iran

**Keywords:** Electrochemistry, Materials chemistry

## Abstract

This study conducted an evaluation of the corrosion behavior of an aluminum alloy utilized in the Isfahan Miniature Neutron Source Reactor (MNSR). The component analyzed, dry channel (DC), had been exposed to radiation for 12 years in a water environment within the reactor pool since its installation. To determine the effect of radiation on the corrosion of the LT-21 aluminum alloy used in the DC, different parts of the pipe were sampled and various tests were performed. These tests included mechanical strengths (impact, and micro-hardening), XRD, TEM, SEM–EDS, and potentiodynamic polarization (PDP). The parameters measured included corrosion potential, corrosion rate, changes in microscopic structure, and mechanical properties of the aluminum alloy along the entire length of the DC. The neutron and gamma dose distribution along the height of the DC, which was 540 cm, was calculated to determine the correlation between the dose distribution and observed corrosion. The study found that the corrosion mechanisms were complex and resulted from the simultaneous presence of the DC in the pool water and radiation from the reactor core. The observed results are presented and discussed in this study.

## Introduction

Corrosion poses a significant threat to numerous industries where it can result in catastrophic consequences^1^. To combat this issue, physical barriers such as paints, varnishes, and organic or inorganic films are often used to coat metal surfaces^2–6^. Additionally, inhibitors can be added to the corrosive medium to control corrosion^7^. Another approach is to use corrosion-resistant metals like aluminum. Aluminum and its alloys are widely used in various industries due to their exceptional properties, including high strength, reduced density, ease of formation and recycling, and corrosion resistance. These alloys have found extensive applications in aerospace, surface coating, marine, shipbuilding industries, and advanced nuclear reactors^8^. Overall, the use of these methods and materials is crucial in preventing corrosion-related damage and ensuring the safety and longevity of industrial equipment and infrastructure.

The utilization of aluminum alloys in nuclear reactors, particularly in research reactors, has gained significant attention due to its outstanding physical properties and remarkable corrosion resistance, particularly in water. Despite the low neutron capture of aluminum, its characteristics have made it an ideal material for various components of reactor containment buildings. These components include fuel cladding, guide tubes, thermal insulations, ladders, scaffoldings, and the containment buildings themselves. The exceptional properties of aluminum alloys have projected them as a choice material for these critical applications^9^. Specifically, the AA-6061-T6 alloy is frequently utilized for core structures and fuel cladding of research reactors, such as Materials Testing Reactors (MTRs), Training, Research, Isotopes, General Atomics Reactors (TRIGAs), and Miniature Neutron Source Reactors (MNSRs)^10–12^. The safe operation of research reactors is of utmost importance, and the study of aluminum alloy corrosion plays a crucial role in ensuring this safety. The aqueous coolant used in these reactors causes corrosion of aluminum alloys, leading to the formation of an aluminum hydroxide film on their surface. However, the thermal conduction of this film is considerably low (~ 2 W/m/K), which can degrade the heat exchange between the core components and the coolant. This, in turn, could result in a local overheating of core components. To address this issue, extensive tests were performed on aluminum alloy corrosion in MTRs, as documented in the literature^12–18^. It is imperative to continue such research to maintain the safe and efficient operation of research reactors^19^.

In general, aluminum is highly susceptible to oxidation, and its ability to resist corrosion is heavily reliant on the formation of a passive oxide film on the alloy. In neutral solutions, radiation may contribute to the formation of thicker oxide films on the alloy, thereby preventing aggressive ions from penetrating through and causing damage. However, radiation exposure can compromise the integrity of this film, making it easier for aggressive ions in a solution to bypass it and affect the underlying alloy matrix. Ionizing radiation can create free radicals and other reactive species that can damage the protective oxide layer on the surface of the aluminum alloy, leading to increased corrosion. Neutron irradiation can also induce the production of hydrogen atoms within materials, which can contribute to hydrogen-induced cracking or corrosion. It is important to consider the potential effects of radiation on aluminum corrosion resistance when designing and using this material in various applications. A thorough understanding of the underlying mechanisms involved can help ensure the long-term durability and reliability of aluminum-based products.

The utilization of aluminum alloy as a containment material exposes it to various ionizing radiation, which can have an impact on the alloy's corrosion behavior. Numerous studies have been conducted to investigate the effect of ionizing radiation on the corrosion behavior of alloys^19–23^. However, there is still much debate among researchers regarding the impact of radiation on material corrosion. Some argue that ionizing radiation does not affect the corrosion behavior of aluminum alloys in water^24,25^, while others hold a contrary view that irradiation accelerates the corrosion of aluminum alloys^26–28^.

In a study conducted by Simnad et al.^24^, it was found that the presence of γ-radiation has a positive impact on the corrosion rate of aluminum in water at room temperature. Krenz^25^ investigated the corrosion of aluminum in a water-cooled reactor with alloying materials like nickel, iron, and silicon for several hours at a high temperature. The results showed that irradiation had only a negligible effect on the corrosion of aluminum. These findings claimed on the potential benefits of γ-radiation in reducing the corrosion rate of aluminum, particularly in water-based environments and under short irradiation time and dose. However, it has been reported in the literature that radiation may accelerate the corrosion of aluminum. Studies conducted by Borasky et al.^26^ revealed that the corrosion rate of aluminum alloys increased with an increase in radiation intensity during hot water irradiation in a reactor. Similarly, Stobes et al.^27^ observed that reactor radiation had a similar effect on the corrosion of aluminum alloy. Gamma irradiation has also been found to accelerate the development of aluminum oxide on the alloy surface, as stated by Kanjana et al.^28^. This is due to the increased density of oxide with extended irradiation and dose time. These findings suggest that caution should be exercised when using aluminum in environments where radiation exposure time and dose is a concern.

Further research is needed to fully understand the relationship between ionizing radiation and the corrosion behavior of aluminum alloys. Given the importance of this material in containment applications, a comprehensive understanding of its behavior under various conditions is crucial.

The LT-21 aluminum alloy, a Chinese alloy with properties similar to the aluminum series 6000X, is utilized in the Isfahan MNSR as fuel clad, inner and outer irradiation sites, dry channel (DC), and reactor tank materials^29,30^. Despite the stability of aluminum alloy under MNSR operation conditions (pH 6, T ~ 38°C, and atmospheric pressure), the ionization radiation caused corrosion of this material. This component, known as a DC, is essentially an aluminum tube with a closed bottom that was utilized for irradiating bulk objects outside the reactor core. The MNSR reactor's DC was in use for 12 years before holes were discovered in its body, necessitating its replacement. Due to its immersion in the pool water, demineralized light water, and exposure to varying radiation doses at different heights, the DC recorded unique and valuable information.

In this study, the DC was divided into six zones at different heights from the reactor's core, and the amount of dose received by each zone was calculated using Monte Carlo simulation and reactor operation data. Surface inspection, mechanical tests, electrochemical analyses, microstructural studies with SEM–EDS and TEM, and XRD were performed on each zone. The results revealed distinct behaviors at different heights of the DC and provided valuable insights into the long-time effects of neutron and gamma irradiations on aluminum parts. The findings can be utilized to design and construct new research reactors and estimate the lifetime of aluminum parts in working reactors. Overall, this study presents significant information related to the impact of radiation on reactor components and highlights the importance of regular inspections and maintenance to ensure safe and efficient reactor operation. These findings are presented and discussed in detail.

## Results and discussions

**Figure 1:**
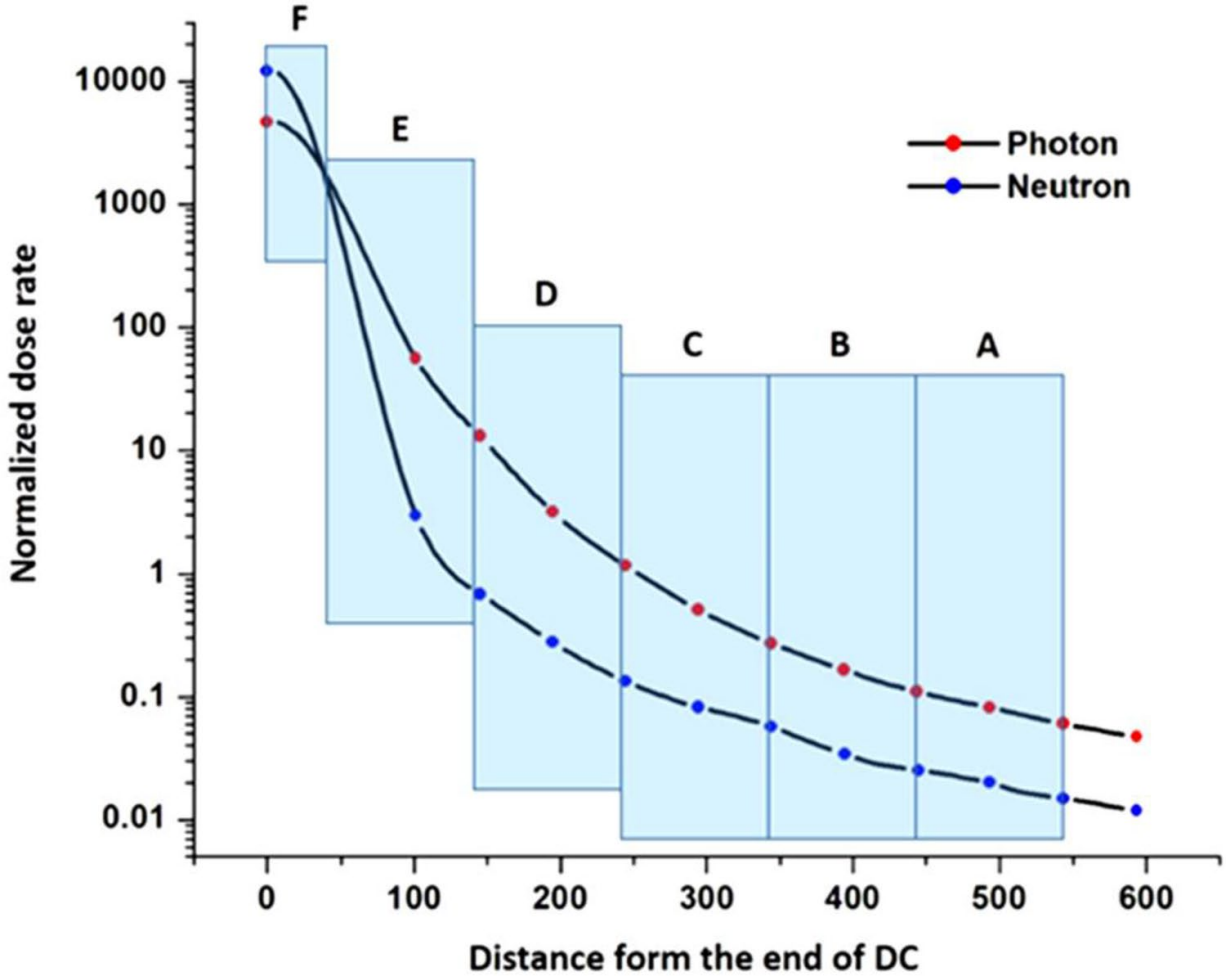
Variation of radiation of neutron and gamma-ray in different zones of the DC.

The operational history of the Isfahan MNSR over, for 12 years has been documented in Table 1. During this time, the neutron dose received on the surface of different parts of the DC varied depending on the distance from the reactor core. Figure 1 illustrates the changes in neutron and photon dose rates across different zones at the DC. The radiation levels were not uniform across all zones, with the highest dose being received in zone *F*, which was situated closest to the reactor core.

**Table 1:** The functional history of Isfahan MNSR operation during 12 years.

Years	First trimester (kWday)	Second trimester (kWday)	Third trimester (kWday)	Fourth trimester (kWday)	Total (kWday)
1	10.17361	12.70833	11.31944	10.76389	44.96528
2	8.923611	15.27778	3.427083	16.49306	44.12153
3	7.291667	68.05556	37.84722	48.61111	161.8056
4	74.65278	61.11111	42.36111	39.58333	217.7083
5	28.125	72.56944	32.67361	34.72222	168.0903
6	31.49306	21.28472	20.76389	32.22222	105.7639
7	11.59722	15.59028	21.5625	16.31944	65.06944
8	20.10417	13.71528	9.756944	14.375	57.95139
9	16.11111	13.78472	26.14583	26.80556	82.84722
10	10.10417	9.097222	28.09028	9.131944	56.42361
11	12.04861	10.20833	9.930556	4.305556	36.49306
12	6.041667	3.784722	2.527778	4.965278	17.31944
13	0.75	2.09375	1.565972	21.97917	26.38889
Total of 12 years					1084.948

The significance of light water as a moderator, coolant, and biological shield is emphasized by the data presented in Table 1 and Fig. 1. The variation in radiation levels across different zones highlights the varying impact of radiation on the DC and potentially harmful levels of radiation. By comprehending the distribution of radiation levels across different zones, researchers and engineers can formulate more effective strategies for mitigating potential risks and minimizing the impact of accidents or incidents that may occur.

**Figure 2:**
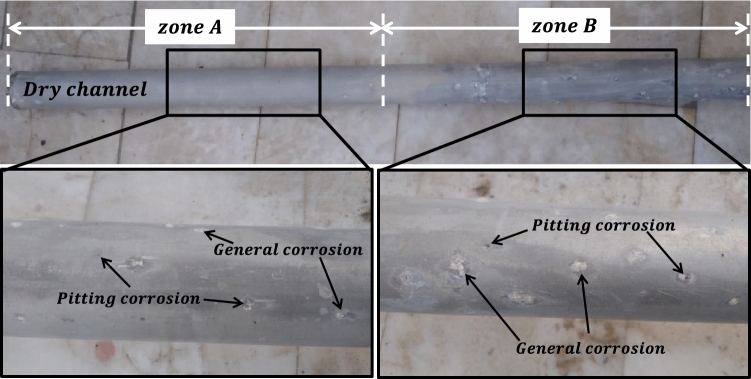
Surface images of the two different zones of DC.

Figure 2, the surface images of the DC are presented, which depicts the extensive corrosion that occurred after 12 years of immersion in the reactor's water pool and radiation exposure. Notably, the surface of the DC exhibits simultaneous pitting and uniform corrosion, which is visible to the naked eye. Inspection of the DC's surface revealed that the area with the highest degree of pitting corrosion was zone *B*, which caused damage to the surface and resulted in water leakage through the holes created inside it. These findings highlight the importance of regular maintenance and monitoring of equipment used in high-risk environments to prevent such corrosion from compromising their functionality and safety.

The effects of neutron radiation on the mechanical properties of aluminum alloys used in Isfahan MNSR reactor have been studied extensively. Mechanical properties calculated from typical stress–strain curves are provided in Table 2. Also, fracture impact energy and micro-hardness are shown in Tables 3, and 4, respectively. The results show that the yield strength of the zones increases as one moves away from the core of the reactor, while elongation decreases significantly up to 4.2%. The ultimate tensile strength and elongation are also decreased significantly in zone *C* compared to other zones. Upon visual inspection, it became apparent that the number of holes and pits in zone *C* exceeded that of the other zones. These holes and pits serve as prime locations for stress accumulation and crack propagation. Consequently, the cracks experienced accelerated growth from the onset of the test, leading to the premature failure of the sample. As a result, the elongation observed in zone *C* appeared to deviate significantly from that of the other zones. The amount of fracture impact energy decreases as one moves closer to the core (zone *F*), reaching about 6.8 J. However, the average micro-hardness measured for different zones shows no significant change (Table 4).

**Table 2:** Mechanical properties calculated from typical stress–strain curves.

Sample	Yield strength (MPa)	Ultimate tensile strength (MPa)	Elongation (%)
A	136	218	25.6
B	138	228	30.8
C	128	183	5.4
D	144	226	35.7
E	141	230	30.8
F	150	160	4.2

**Table 3:** Fracture energy calculated from impact test.

Sample	Impact energy (J)
A	7.9
B	9.0
C	7.7
D	9.2
E	7.9
F	6.8

**Table 4:** Micro-hardness of different zones.

Sample	Hardness 1 (MHV 0.3)	Hardness 2 (MHV 0.3)	Hardness 3 (MHV 0.3)	Hardness 4 (MHV 0.3)	Hardness 5 (MHV 0.3)	Average of hardness (MHV 0.3)
A	88.1	82.2	85.9	92.6	87.1	87.2 ± 3.8
B	82.2	84.4	84.3	81.7	80.0	82.5 ± 1.9
C	79.5	88.4	86.3	82.6	88.5	85.1 ± 4.0
D	91.2	91.0	87.0	98.6	94.9	92.5 ± 4.4
E	91.2	86.4	88.1	89.5	82.4	87.5 ± 3.4
F	89.0	92.5	85.7	88.5	84.8	88.1 ± 3.0

Neutron irradiation causes damage to aluminum alloys through displacement formation by fast neutrons and transmutation damage by both thermal and fast neutrons. The relative contribution of these damage mechanisms and their impact on mechanical properties depend on the alloy composition, thermal-to-fast neutron fluence ratio, irradiation temperature, and other irradiation conditions. With increasing irradiation dose, dislocation loops also grow and contribute to the increase in network dislocation density, leading to irradiation hardening and embrittlement. The dislocation density in irradiated metals evolves toward a saturation value with increasing dose, at which point transmutation-produced silicon plays a dominant role in contributing to irradiation hardening^31^.

**Figure 3:**
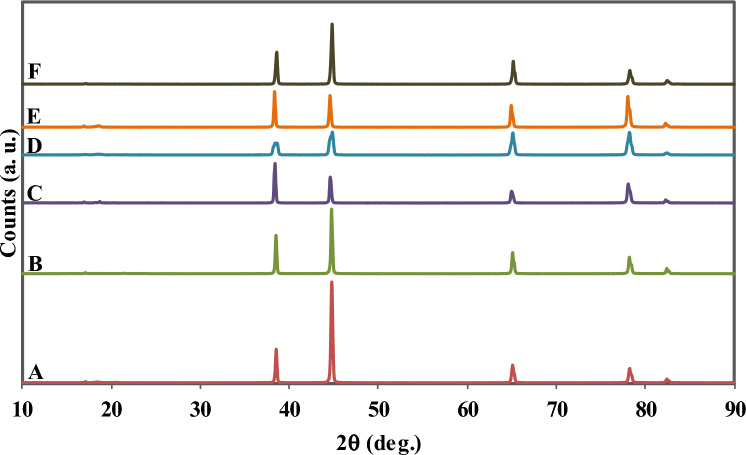
The XRD patterns samples from *A*, *B*, *C*, *D*, *E*, and *F* zones after 12 years of irradiation.

To establish a relationship between irradiation dose and dislocation density, XRD analysis was performed on samples from zone *A* to *F*. Figure 3 displays the XRD patterns obtained from the analysis, and Table 5 presents the calculated dislocation densities based on the Williamson-Hall equation and observed crystallite size. The results indicate that dislocation density in zones *D*, *E*, and *F* increased significantly compared to other zones. This finding is consistent with the observed hardness data presented in Table 4.

**Table 5:** Crystallite size, micro strain, and density of dislocations calculated for different zones.

Zone	Crystallite size (D/nm)	Micro strain(ε)^a^	Density of dislocation^b^ ($\rho$/nm^2^)
A	876	1.5 e−04	1.303 e−06
B	928	1.01 e−04	1.161 e−06
C	825	3.25 e−04	1.469 e−06
D	469	7.96 e−04	4.546 e−06
E	588	5.86 e−04	2.892 e−06
F	680	2.75 e−04	2.162 e−06

^a^Calculated by using $\frac{\beta \mathrm{cos}\theta }{\lambda }=4\varepsilon \frac{\mathrm{sin}\theta }{\lambda }+\frac{K}{D}$ equation.

^b^Calculated by using $\rho =\frac{1}{{D}^{2}}$ equation, where *D* is crystallite size from the XRD patterns.

**Figure 4:**
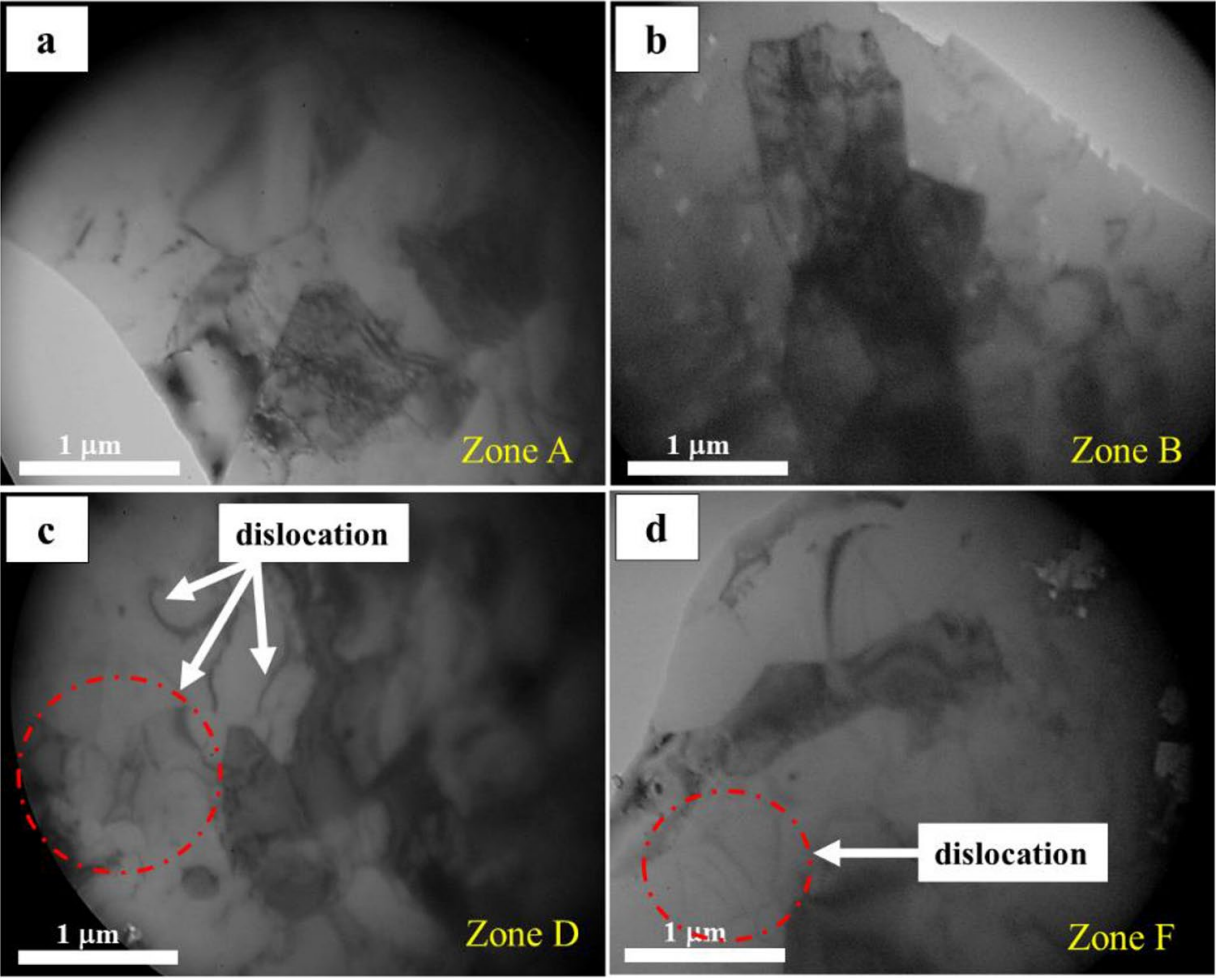
The TEM images of samples from *A*, *B*, *D*, and *F* zones after 12 years of irradiation.

In order to substantiate the occurrence of dislocation formation over extended periods of irradiation, TEM images of certain zones were conducted, as depicted in Fig. 4. The results indicate that an increase in dose rate in zones *D* and *F* led to a rise in dislocation density, which consequently caused the formation of small-sized subgrains. It was observed that a decrease in the size of the subgrains corresponded to an increase in hardness. These findings provide compelling evidence for the influence of irradiation on the microstructure of the DC material.

As can be conclude, the increase in radiation dose reduces elongation and fracture energy, as shown in Tables 2 and 3. Transmutation of silicon in aluminum alloys nucleates as amorphous silicon particles in the matrix, associates with irradiation-induced voids, and decorates existing Mg_2_Si precipitates. However, the contribution of this mechanism to irradiation hardening is low because deformation caused by the shearing of soft silicon particles produces little strain hardening. Similarly, the hardening contribution from an increase in the size of existing Mg_2_Si precipitates due to Si decoration is also low.

However, the study shows that neutron radiation causes significant changes in the mechanical properties of aluminum alloys used in the Isfahan MNSR reactor. The increase in radiation dose reduces elongation and fracture energy, while the micro-hardness remains unaffected. Further studies like surface imaging and elemental analysis are needed to understand the underlying mechanisms and develop strategies to mitigate the effects of neutron radiation on aluminum alloys.

**Figure 5:**
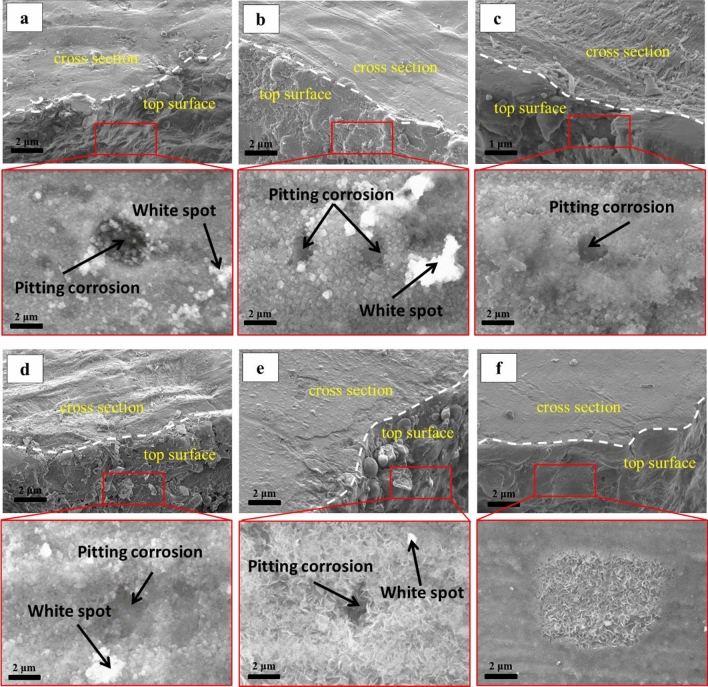
The cross-sectional SEM micrographs and corresponding top surface SEM images of (**a**) *A*, (**b**) *B*, (**c**) *C*, (**d**) *D*, (**e**) *E*, and (**f**) *F* zones after 12 years of irradiation.

The cross-sectional SEM micrograph in Fig. 5 depicts the development of uneven white spots on the surface of all samples. Interestingly, the structure of the oxide layer formed on the surface varied across different zones. As the distance from the core of the reactor increased, the structure of the oxide layer became more crystalline. On the other hand, the oxide layer formed near the core of the reactor was more amorphous (zone *F*). This indicates that higher radiation doses near the core of the reactor had a significant influence on the morphology of the oxide layer. The authors suggest that the product formed was alumina Al(OH)_3_.

Furthermore, increasing the distance to the reactor core led to an increase in both the size and number of white spots. Notably, these white spots were not visible in zone *F*. This observation highlights the effect of absorbed radiation dose from the MNSR core on the properties of samples. Overall, the SEM micrograph provides valuable insights into the structural characteristics of the oxide layer formed on the surface of the samples.

**Figure 6:**
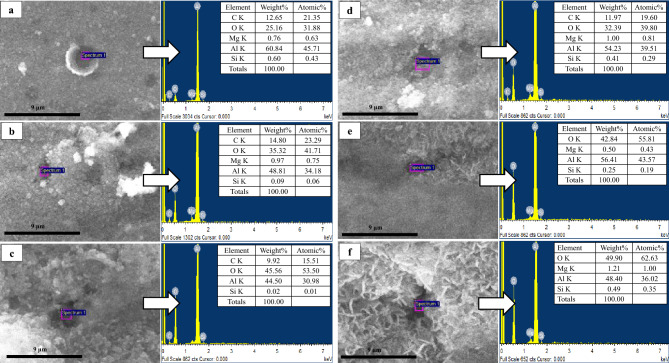
The top surface SEM micrographs and EDS spectrums of the oxide layer of (**a**) A, (**b**) B, (**c**) C, (**d**) D, (**e**) E, and (**f**) F zones after 12 years of irradiation.

The analysis presented in Figs. 2 and 5 revealed the areas that have been affected by pitting corrosion, which were indicated by arrows. Interestingly, it was observed that the number of holes formed in zone *B* was significantly higher compared to the other zones. To further investigate the composition of corrosion layer, an elemental analysis was conducted and the results were presented in Fig. 6. The analysis showed that the product formed on the surface was composed of Al, O, Si, Mg, and C. However, it is worth noting that the presence of carbon in the composition could be attributed to contamination during the sample preparation process.

Furthermore, the presence of Si in the composition could be attributed to the transformation of Al into Si due to thermal neutron following reactions (1) and (2).

1 $$ {}_{{13}}^{{27}} Al + {}_{0}^{1} n\mathop  \to \limits^{{(n,\gamma )}} {}_{{13}}^{{28}} Al $$

2 $${}_{{13}}^{{28}} Al\to {}_{{14}}^{{28}}Si+{e}^{-}$$

During the corrosion of Mg-containing Al alloy, a Mg release in solution was observed. In an aqueous medium at a pH of 6, the stable form of oxidized Mg was the aqueous ion Mg^2+^, according to the corresponding Pourbaix diagram.

In summary, observed results shed light on the factors contributing to pitting corrosion in Al alloys. The results suggest that zone *B* is more susceptible to pitting corrosion due to its elemental composition.

**Figure 7:**
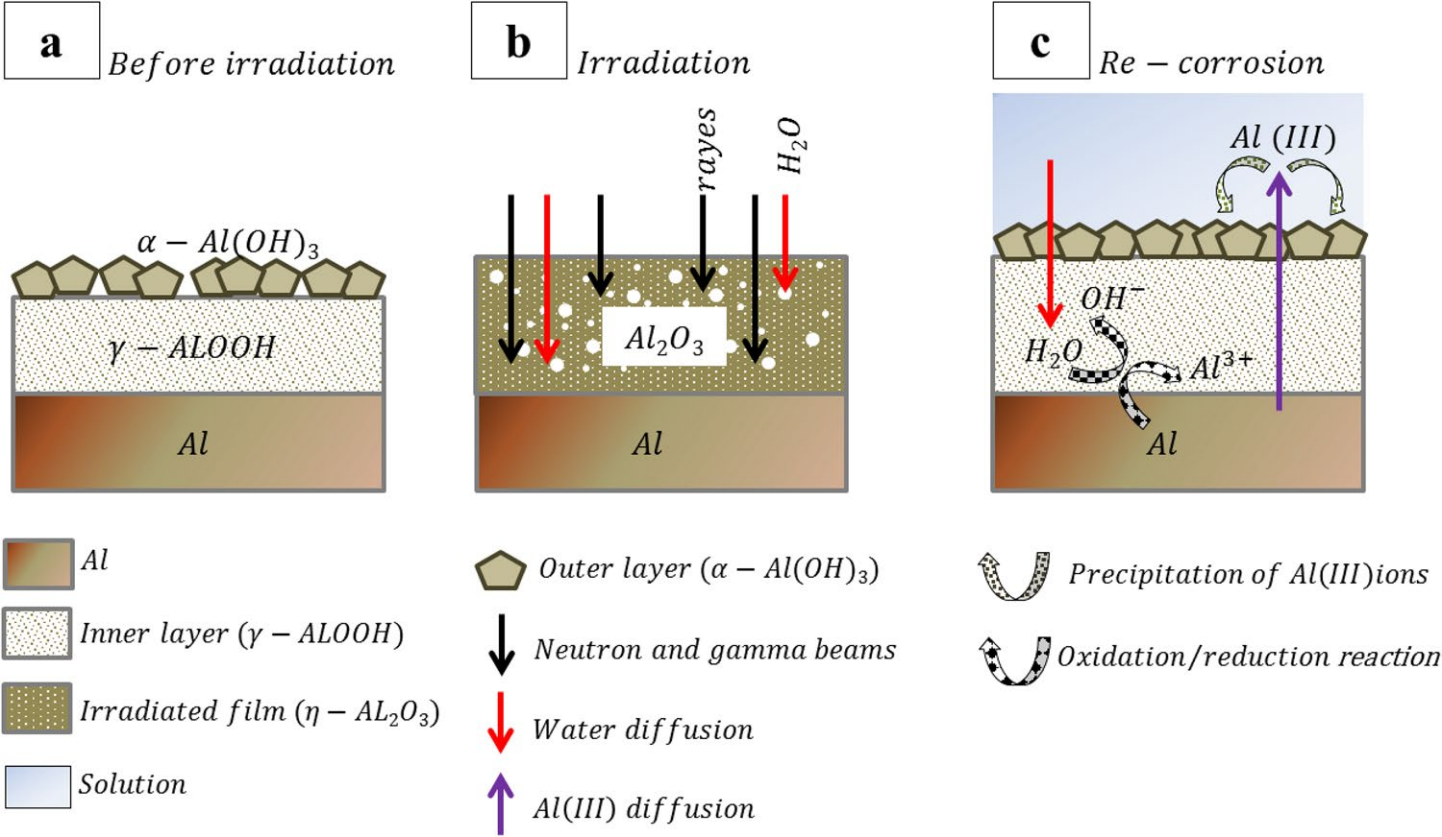
Schematic of the different steps of the proposed mechanism of aluminum corrosion during the long-time irradiation in the MNSR reactor.

The demineralized light water used as a coolant in the Isfahan MNSR reactor have been observed to cause corrosion of the DC due to the inherent corrosive nature of water. This results in the formation of aluminum hydroxide on the surface of the DC. In the absence of irradiation, this oxidation reaction is accompanied by the reduction of water and dioxygen, leading to the production of OH hydroxide groups. Some of the oxidized aluminum reacts with the OH hydroxide groups to form boehmite (γ-AlOOH) at the hydroxide-metal interface, while the rest diffuses through the film and is released as ion Al(III) in the solution. This ion then precipitates on the sample surface to form bayerite (α-Al(OH)_3_), the outer layer (Fig. 7a).

During long-time irradiation, changes in microstructure are observed as the formed aluminum hydroxide is dehydrated (Fig. 7b). Due to the non-protective quality of this irradiated film, the matrix is oxidized and Al is released in solution (Fig. 7c). A new outer layer is formed by the precipitation of Al ions in solution on the sample surface. After irradiation, during the re-corrosion of the sample surface, the irradiated film becomes the new inner layer. Alumina obtained from dehydrated aluminum hydroxide is rehydrated by water to form boehmite, the crystalline phase of the inner layer. The proposed global reactions are

3 $$2Al\left(OH\right)\to {Al}_{2}{O}_{3}+{H}_{2}O$$

4 $${2Al(OH)}_{3}\to {Al}_{2}{O}_{3}+{3H}_{2}O$$

However, the morphology of the formed oxide is affected by radiation, with an increase or decrease in radiation leading to changes in morphology. As one approaches the core of the reactor and the radiation dose increases, the morphology of the oxide becomes more amorphous. Increasing the amount of radiation leads to more stable alumina oxide on the surface, with neutron irradiation leading to more re-corrosion and formation of alumina hydroxide and boehmite on the surface. At room temperature, the oxide product generated from the oxidation of Al in water is commonly found in the form of amorphous alumina or crystalline aluminum hydroxide (AlOOH), boehmite.

During the irradiation period, also radiolysis occurs on the surface of aluminum. This process results in the formation of hydrated electrons, which react with water molecules to produce various species, including e_aq_^−^, H_3_O^+^, H^⋅^, H_2_, ^⋅^OH, and H_2_O_2_ (Eq. 5).

5 $$\mathrm{H}_{2}\mathrm{O}\to \mathrm{e}_{\rm aq}^{-},\mathrm{ H}_{3}\mathrm{O}^{+},\mathrm{ H}^{\cdot} ,\mathrm{ H}_{2}, ^{\cdot} \mathrm{OH},\mathrm{ H}_{2}\mathrm{O}_{2}$$

As the radiation dose increases, more hydrogen is produced on the surface, leading to the formation of bubbles. The explosion of these bubbles removes and destroys the oxide layer on the surface, resulting in an increase in the local corrosion rate. As one approaches the reactor's core, the production of hydrogen bubbles also increases, resulting in more localized corrosion and a higher corrosion rate. Consequently, radiolysis and the resulting hydrogen production also play a significant role in the corrosion of aluminum surfaces during long-time irradiation.

**Figure 8:**
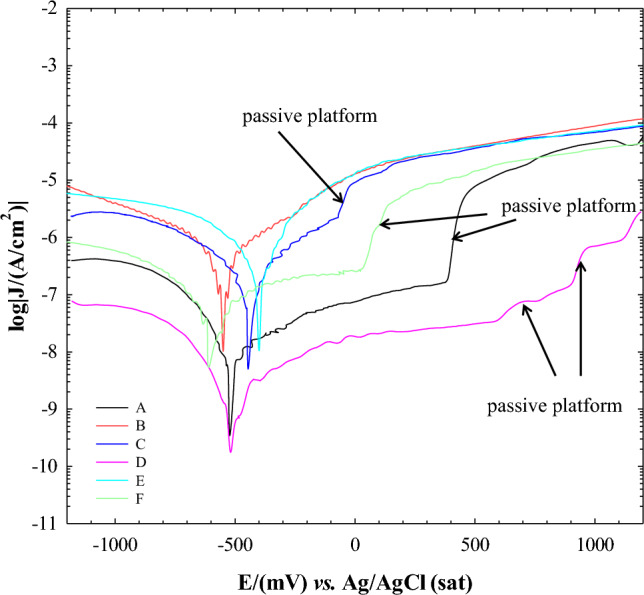
Polarization curves for the A to F zones obtained in water pool solution.

Figure 8 represents the anodic and cathodic polarization curves of the DC samples in different zones (*A* to *F*) in deionized water used in the pool water of the Isfahan MNSR reactor. The corrosion rate (*CR*) using polarization data was calculated through the following equation:

6 $$CR={k}_{1}\frac{{I}_{corr}}{\rho }EW$$

$${k}_{1}=3.27\times {10}^{-3} \frac{\mathrm{mm}}{\mathrm{\mu A\,cm\,year}}$$

$$\rho = 2.70 \mathrm{g}/{\mathrm{cm}}^{3}$$

$$EW = 9.01 \mathrm{g}$$

Table 6 shows the relevant parameters including corrosion current density (*j*_*corr*_), anodic and cathodic Tafel slopes (*β*_*a*_, *β*_*c*_), corrosion potential (*E*_*corr*_), polarization resistance (*R*_*p*_), corrosion rate (*CR*), pitting corrosion potential (E_pit_), and resistance of the oxide layer (∆E_Pit_ = E_pit_ − E_corr_). According to the polarization curves in Fig. 8, the behavior of the DC samples is different due to surface morphology during long-time exposure to irradiation in the water pool of the Isfahan MNSR reactor. As can be seen, the highest corrosion current was related to zone *B* (143.6 × 10^–3^ μA/cm^2^).

**Table 6:** Information obtained from the PDP of A to F zones in Fig. 8.

Zone	J_corr_ (μA/cm^2^)	E_corr_ (mV)	*B*_*a*_ (mV)	*B*_*c*_ (mV)	Rp (Ω/cm^2^)	CR (mm/year)	E_Pit_	∆E_Pit_
A	3.446 × 10^–3^	−520	826.3	210.9	1.6079 × 10^7^	37.7 × 10^–6^	375	895
B	143.6 × 10^–3^	−549	330.3	338.9	4.4965 × 10^5^	1.57 × 10^–3^	−250	299
C	4.095 × 10^–3^	−444	201.3	246.4	1.2258 × 10^6^	44.7 × 10^–6^	−73	371
D	1.260 × 10^–3^	−514	398.6	189.5	4.0772 × 10^7^	13.8 × 10^–6^	570	1084
E	97.00 × 10^–3^	−399	193.9	335.6	6.0418 × 10^5^	1.06 × 10^–3^	–	–
F	20.54 × 10^–3^	−592	322.7	253.5.2	2.6031 × 10^6^	2.25 × 10^–4^	7	586

The results presented in Table 6 demonstrate that the Al LT21 alloy used in this reactor has shown improved corrosion resistance in zones *A* to *F* when compared to the non-irradiated alloy. The uniform corrosion rate of the non-irradiated alloy was reported as 5.0 × 10^–3^ mm/y, whereas the corrosion rate of the irradiated alloy was lower in the aforementioned zones. This observation suggests that neutron radiations have contributed to the increased corrosion resistance of the alloy in deionized water environments during long-time irradiation.

Further analysis of the corrosion rates in different zones that received varying doses of radiation revealed that area *B*, located at a height of one meter above the pool water level or a distance of 3 m from the MNSR core, had the highest corrosion rate. The obtained data are consistent with visual inspection and hole counting, indicating that area *B* has the highest level of pitting corrosion. Pitting corrosion is a type of corrosion that occurs locally on the surface of a material, unlike uniform corrosion which affects the entire surface. It is characterized by the formation of corrosion holes in areas where the resistant layer has lost its protective properties and dissolved. One of the most important results in electrochemical tests is the determination of the potential of pitting corrosion (E_pit_), which is related to the germination and growth of corrosion pits.

The resistance to pitting corrosion is directly related to the pitting corrosion potential (E_pit_) and the width of the resistant zone (∆E_Pit_). Based on the results presented in Table 6, it is evident that zone *D* has the highest E_pit_ and ∆E_Pit_, while the lowest values belong to zone *B*. However, it should be noted that in sample *E*, due to the high rate of active dissolution at the beginning of the anodic scanning process, no specific corrosion potential was observed. Zone *C* has a similar pitting corrosion behavior to zone *B*, but with higher values of E_Pit_ and ∆E_Pit_, indicating better resistance to pitting corrosion. Zone *A* has a lower resistance to pitting corrosion than zone *D* due to its lower E_Pit_ and ∆E_Pit_ values. Zone *F* has better pitting corrosion resistance than zones *B* and *C* but less than zones *A* and *D*, based on its E_Pit_ and ∆E_Pit_ values. The cumulative dose received from zone *A* to *F* showed that with an increase in dose from zone *A* to *B*, the resistance to pitting corrosion decreases significantly. However, with an increase in dose from zone *B* to *D*, there is a gradual increase in pitting corrosion resistance until it reaches its maximum level in zone *D*. Further increase in dose in zone *D* causes a decrease in pitting corrosion resistance in zone *E* and then an increase again in zone *F*. Visual inspections reveal that the corrosion product density of the radiation sample in zone *B* was lower compared to other zones (Fig. 2). This indicates that the intensity of radiation dose resulted in defects in DC, particularly on the surface layer. Furthermore, zone *B*'s ability to form a dense protective passive layer was less than other zones due to its location in the tow-phase area, making it more susceptible to pitting and uniform corrosion. Consequently, the corrosion current was higher in this zone.

## Conclusion

In this research, the corrosion behavior of aluminum alloy used in the Isfahan MNSR has been evaluated. For this purpose, one of the aluminum components of the reactor has been used, which has been under radiation for 12 years since the installation of the reactor in the water environment inside the reactor pool. The results showed that pitting and uniform corrosion occurred simultaneously due to the presence of a DC in the water pool and radiations on the surface. Although the rate of uniform corrosion for Al alloy in a water environment is typically low (about 5 × 10^–3^ mm/year), the pitting corrosion was considered the dominant mechanism in this study. It was also observed that the location of zone *B* in the tow-phase area, coupled with the lack of formation of a dense protective passive layer, resulted in a significantly higher corrosion current compared to other zones (143.6 × 10^–3^ μA/cm^2^). Generally, the defects and grain boundaries facilitated oxygen infiltration and diffusion of Al, Si, and Mg atoms. The paths of defects were larger than those of grain boundaries, leading to changes in the corrosion behavior of the DC in each zone with varying radiation doses. The corrosion mechanism can be explained by the fact that grain boundaries have weak corrosion resistance, and the diffusion of Al atoms occurs primarily along these boundaries in a water pool. The Al atoms combine with oxygen atoms to form Al_2_O_3_, which restrains the diffusion of Si and Mg atoms, leading to intercrystalline Al diffusion into the water pool. This results in the generation of some Al_2_O_3_ in the intercrystalline region, which grows further. Additionally, some Si and Mg atoms react with each other along with grain boundaries and generate Mg_2_Si precipitates. Neutron radiation on the surface led to the formation of Mg_2_Si precipitates in the grain boundaries of the DC, with increasing radiation doses resulting in more deposition of these precipitates in the grain boundaries. The presence of these precipitates as impurities affected the corrosion current, causing an increase inside the core of the reactor (E and F zones). On the other hand, the formation of hydrogen bubbles and local explosions on the surface of the oxide layer in zone *F* causes local removal of oxides, making it susceptible to corrosive ions. The study concludes that despite being closer to the reactor core, zones *E* and *F* have less corrosion resistance than zone *D* due to their susceptibility to uniform corrosion. The increase in dislocations with increasing dose leads to the formation of sub-boundaries and smaller grains, which increases surface hardness. While, the presence of hydrogen bubbles on the surface leads to stress concentration and crack propagation, resulting in lower hardness and fracture energy zones *E* and *F* compared to zone *D.*

In conclusion, the contribution of neutron particles and gamma rays must be considered in interpretation of the observed results for radiation corrosion of the DC in the water pool of reactor. It is well known that the gamma ray is a photon and an electromagnetic radiation, but neutron is a particle, and their interactions with materials is differ. Neutrons can collide with atomic nuclei of Al, and cause displacement of Al atoms within the DC. This displacement of Al atoms lead to structural changes, defects, or the formation of new phases within the DC, which affected on its corrosion resistance. Neutron irradiation also induce the production of hydrogen atoms within Al, which can contribute to hydrogen-induced cracking or corrosion. On the other hand, gamma rays can penetrate materials deeply and ionize atoms in their path. In terms of corrosion of the DC, gamma rays induced chemical reactions in water, leading to the formation of corrosive species such as free radicals or reactive oxygen species from water radiolysis. Consequently, the gamma rays resulted in localized corrosion, pitting, or uniform degradation of the DC. In summary, gamma rays primarily induce chemical reactions, while neutron rays cause structural changes and displacement of atoms in the DC. Both types of radiation contributed to corrosion of the DC in aqueous environment through different mechanisms. However, these findings highlight the importance of considering multiple factors when analyzing corrosion behavior, particularly in complex environments such as those found in nuclear reactors. Furthermore, our findings can be beneficial for designing and maintaining similar reactors.

## Materials

**Figure 9:**
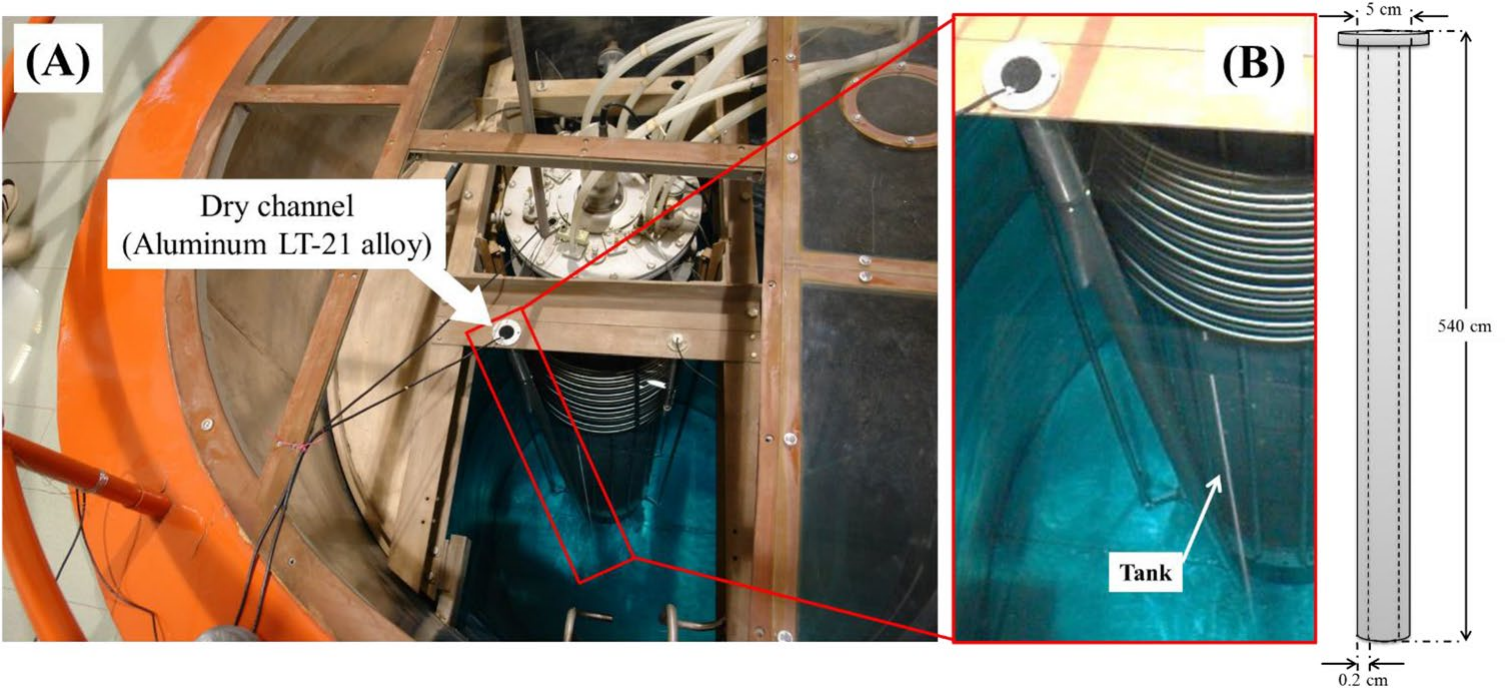
(**a**) Location and (**b**) dimensional of the DC in Isfahan MNSR reactor.

The Isfahan MNSR has been in operation since 1994, serving as a 30 kW research reactor for a variety of applications such as neutron activation analysis, radioisotope production, and nuclear engineering research^29,30,32–35^. This tank-in-pool type reactor utilizes demineralized light water as a moderator, coolant, and biological shield, with a conductivity of less than 5 µS/cm for the reactor pool and 2 µS/cm for the reactor tank. The majority of the reactor's components, including the tank, fuel clads, and guide tubes, are constructed from aluminum. The DC, or guide tube for irradiation outside the reactor core, is also made of aluminum and is easily accessible for installation or removal. However, after 12 years of use, the first DC suffered corrosion and perforation, rendering it unusable. Technical and visual inspections revealed damage to several segments of the surface region. Table 7 displays the chemical composition of the LT-21 Aluminum alloy (series 6000X) used in the first DC, while Fig. 9 illustrates its location and dimensions (540 cm and 5 cm) within the Isfahan MNSR reactor.

**Table 7:** Chemical composition of the LT-21 aluminum alloy.

Al	Mg	Si	Cu	Ti	Fe	Ni	Zn	Mn	B
Base	0.45–0.9	0.6–1.2	0.01 $\le$	0.01 $\le$	0.2 $\le$	0.015 $\le$	0.03 $\le$	0.01 $\le$	0.0002 $\le$

**Figure 10:**
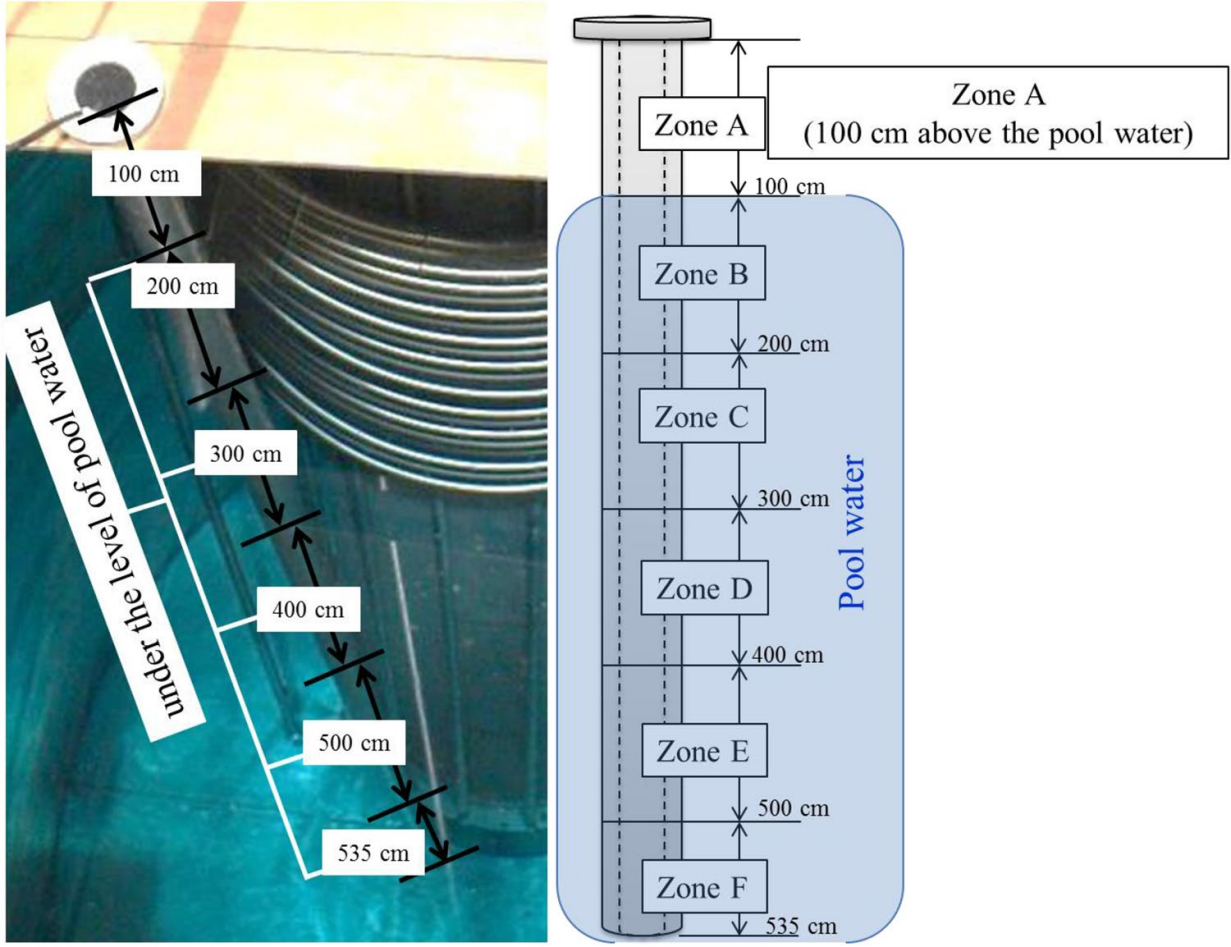
The schematic of different segments of the DC. The numbers represent the depth of the sample relative to the water surface.

The MNSR reactor core is located within the deepest part of the reactor tank and has a cylindrical shape with a diameter and height of 23 cm. The DC, which is responsible for preparing samples for tests, is situated outside the tank and within the pool. Its closed end is at the same level as the core's bottom, and the upper part of the DC pipe is flanged to the upper support of the tank, as depicted in Fig. 9. To investigate the effect of different amounts of radiation dose absorbed in the tube, the DC was divided into six zones from *A* to *F* on the height of the pipe, as shown in Fig. 10. As can be seen from Fig. 10, zone A is above of the pool water and corroded in water vapor. The division was intended to determine the extent of absorbed radiation dose, with area *F*, located closest to the reactor core, receiving the highest amount of absorbed radiation dose, while other sections up to *A* received fewer doses. This approach ensures accurate testing and analysis of samples.

**Figure 11:**
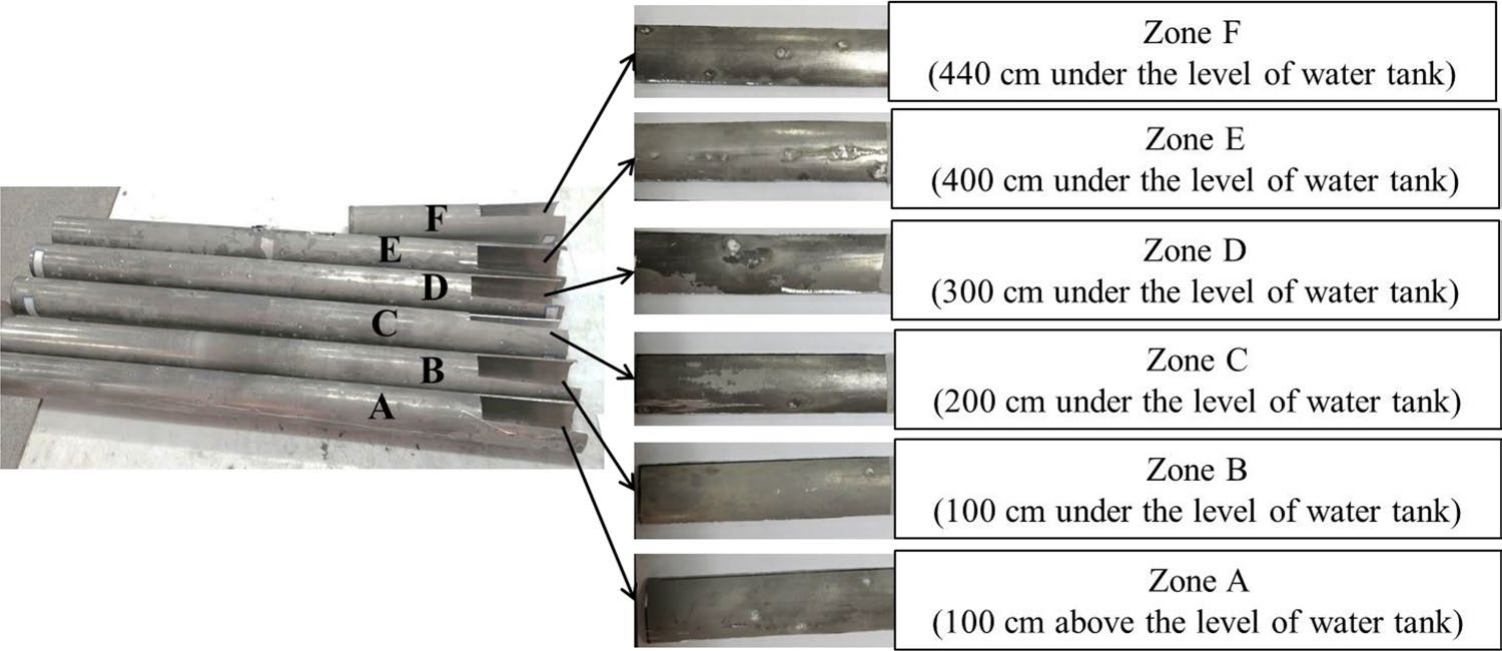
The square-shaped samples from each zone.

In this study, a specimen was cut into a square shape from each zone with dimensions of 200 mm × 50 mm × 2 mm (Fig. 11). Microstructural analysis was conducted using a Philips Scanning Electron Microscope (SEM) coupled with an energy dispersive spectroscope (EDS) sensor. Mechanical properties were evaluated through the mechanical strengths test, impact test, and micro-hardening test, all carried out according to ASTM E384, ASTM E23, and ASTM E8 standards. To study the corrosion behavior of samples, the potentiodynamic polarization (PDP) test in the water pool of the Isfahan MNSR reactor was conducted using the Behpajouh (model of Skydat1) device. A conventional three-electrode cell was used with platinum as the counter electrode, Ag/AgCl (KCl sat) electrode as the reference electrode, and the main sample electrode. OCP measurement was taken after all samples were immersed in the water pool of the Isfahan MNSR reactor for 30 min. The PDP measurement was carried out according to ASTM G5 and ISO 16283 standards, from point potential 1.2 V, more negative than the OCP up to increase suddenly of applied current density. Current density, potential, as well as, slopes of the anode and cathode regions were important parameters for the evaluation of corrosion resistance. The polarization resistance (${P}_{p}$) was calculated by Stern’s Gray’s. The crystal structure of the prepared samples were studied by an X-ray diffractometer (XRD, Bruker Advanced D8 model), using Cu Kα radiation (λ = 1.5406 Å). The microstructural size of the samples was characterized using transmission electron microscope (Philips-EM-208S). Overall, these methods provide a comprehensive understanding of the mechanical and corrosion properties of the samples under study.

## Data Availability

The information that corroborates the discoveries of this investigation can be accessed from the corresponding author upon a rational appeal.

## References

[1] Bagherzadeh M, Ghahfarokhi ZS (2023). Graphene-based 2D materials: Recent progress in corrosion inhibition. Smart Anticorros. Mater..

[2] Bagherzadeh M, Mousavi O, Ghahfarokhi ZS (2020). Fabrication and characterization of a Fe_3_O_4_/polyvinylpyrrolidone (Fe_3_O_4_/PVP) nanocomposite as a coating for carbon steel in saline media. New J. Chem..

[3] Ghahfarokhi, Z. S., Bagherzadeh, M., Yazdi, E. G. & Teimouri, A. Surface modification of graphene-coated carbon steel using aromatic molecules for enhancing corrosion resistance; Comparison between type of aryl substitution with different spatial situations. In *Anti-Corrosion Methods and Materials* (2018).

[4] Yazdi EG, Ghahfarokhi ZS, Bagherzadeh M (2017). Protection of carbon steel corrosion in 3.5% NaCl medium by aryldiazonium grafted graphene coatings. New J. Chem..

[5] Bagherzadeh M, Ghahfarokhi ZS, Yazdi EG (2016). Electrochemical and surface evaluation of the anti-corrosion properties of reduced graphene oxide. RSC Adv..

[6] Bagherzadeh M, Haddadi H, Iranpour M (2016). Electrochemical evaluation and surface study of magnetite/PANI nanocomposite for carbon steel protection in 3.5% NaCl. Prog. Organ. Coat..

[7] Bagherzadeh M, Jaberinia F (2018). Electrochemical study of Monel alloy corrosion in hydrochloric acid solution and pyrrolidine dithiocarboxylate self-assembled monolayers as its corrosion protector. J. Alloy. Compd..

[8] Fathi P, Mohammadi M, Duan X, Nasiri AM (2018). A comparative study on corrosion and microstructure of direct metal laser sintered AlSi10Mg_200C and die cast A360.1 aluminium. J. Mater. Process. Technol..

[9] Howe KJ, Mitchell L, Kim S-J, Blandford ED, Kee EJ (2015). Corrosion and solubility in a TSP-buffered chemical environment following a loss of coolant accident: Part 1–Aluminum. Nucl. Eng. Des..

[10] Pawel R, Yoder G, West C, Montgomery B (1990). The Development of a Preliminary Correlation of Data on Oxide Growth on 6061 Aluminum Under ANS Thermal-Hydraulic Conditions.

[11] Griess J, Savage H, English J (1964). Effect of Heat Flux on the Corrosion of Aluminium by Water. Part IV: Tests Relative to the Advanced Test Reactor and Correlation with Previous Results.

[12] Wintergerst M, Dacheux N, Datcharry F, Herms E, Kapusta B (2009). Corrosion of the AlFeNi alloy used for the fuel cladding in the Jules Horowitz research reactor. J. Nucl. Mater..

[13] Richt A, Knight R, Adamson Jr G (1971). Postirradiation Examination and Evaluation of the Performance of HFIR Fuel Elements.

[14] Farrell K, King R, Jostsons A (1973). Examination of the Irradiated 6061 Aluminum HFIR Target Holder.

[15] Hanson, G., Gribson, G. & Griess, J. *CONF-8811203, Oak Ridge National Laboratory.*

[16] Quaireau SLH (2021). Impact of ion and neutron irradiation on the corrosion of the 6061-T6 aluminium alloy. J. Nucl. Mater..

[17] Kim YS (2020). Aluminum cladding oxide growth prediction for high flux research reactors. J. Nucl. Mater..

[18] Kim YS, Hofman G, Robinson A, Snelgrove J, Hanan N (2008). Oxidation of aluminum alloy cladding for research and test reactor fuel. J. Nucl. Mater..

[19] Deng P, Peng Q, Han E-H (2021). Grain boundary oxidation of proton-irradiated nuclear grade stainless steel in simulated primary water of pressurized water reactor. Sci. Rep..

[20] Srivastava A (2022). Understanding the effect of irradiation temperature on microstructural evolution of 20MnMoNi55 steel. Sci. Rep..

[21] Lu C (2016). Enhancing radiation tolerance by controlling defect mobility and migration pathways in multicomponent single-phase alloys. Nat. Commun..

[22] Deng S-H, Lu H, Li D (2020). Influence of UV light irradiation on the corrosion behavior of electrodeposited Ni and Cu nanocrystalline foils. Sci. Rep..

[23] Zhou W (2020). Proton irradiation-decelerated intergranular corrosion of Ni-Cr alloys in molten salt. Nat. Commun..

[24] Simnad M (1957). Influence of Radiation Upon Corrosion and Surface Reactions of Metals and Alloys.

[25] Krenz F (1957). Corrosion of aluminium–nickel type alloys in high temperature aqueous service. Corrosion.

[26] Borasky R, Mastel B (1955). Surface Structure of Pile Irradiated 2S Aluminum.

[27] Stobbs J, Swallow A (1962). Effects of radiation on metallic corrosion. Metall. Rev..

[28] Kanjana K, Ampornrat P, Channuie J (2017). Gamma-radiation-induced corrosion of aluminium alloy: Low dose effect. J. Phys. Conf. Ser..

[29] Mokhtari J, Dastjerdi MC (2023). Development and characterization of a large thermal neutron beam for neutron radiography at Isfahan MNSR. Nucl. Instrum. Methods Phys. Res. Sect. A Acceler. Spectrom. Detect. Assoc. Equip..

[30] Dastjerdi MC, Mokhtari J, Asgari A, Ghahremani E (2019). A neutron radiography beamline relying on the Isfahan Miniature Neutron Source Reactor. Nucl. Instrum. Methods Phys. Res. Sect. A Acceler. Spectrom. Detect. Assoc. Equip..

[31] Hutagaol AG, Hidayat I, Ajiriyanto MK, Zakaria S (2017). Post-irradiation mechanical properties prediction of Al 6070 of MTR-fuel bundle for cutting process. SINERGI.

[32] Mokhtari J, Faghihi F, Khorsandi J (2017). Design and optimization of the new LEU MNSR for neutron radiography using thermal column to upgrade thermal flux. Prog. Nucl. Energy.

[33] Mokhtari J, Faghihi F, Dastjerdi MC, Khorsandi J (2020). Neutronic feasibility study of using a multipurpose MNSR for BNCT, NR, and NAA. Appl. Radiat. Isotopes.

[34] Moslehi A, Dastjerdi MC, Torkzadeh F, Mokhtari J (2022). Feasibility study of Isfahan MNSR as a calibration thermal neutron source. Nucl. Instrum. Methods Phys. Res. Sect. A Acceler. Spectrom. Detect. Assoc. Equip..

[35] Abbassi Y, Mirvakili SM, Mokhtari J (2022). Development of a fast thermal-hydraulic model to simulate heat and fluid flow in MNSR. Ann. Nucl. Energy.

